# Increased Expressions of ADAMTS-13, Neuronal Nitric Oxide Synthase, and Neurofilament Correlate with Severity of Neuropathology in Border Disease Virus-Infected Small Ruminants

**DOI:** 10.1371/journal.pone.0120005

**Published:** 2015-03-23

**Authors:** Gungor Cagdas Dincel, Oguz Kul

**Affiliations:** 1 Laboratory and Veterinary Health Program, Siran Mustafa Beyaz Vocational School, University of Gumushane, Gumushane, Turkey; 2 Department of Pathology, Faculty of Veterinary Medicine, University of Kirikkale, Kirikkale, Turkey; The Scripps Research Institute, UNITED STATES

## Abstract

Border Disease (BD), caused by *Pestivirus* from the family *Flaviviridae*, leads to serious reproductive losses and brain anomalies such as hydranencephaly and cerebellar hypoplasia in aborted fetuses and neonatal lambs. In this report it is aimed to investigate the expression of neuronal nitric oxide synthase (nNOS), A Disintegrin And Metalloprotease with Thrombospondin type I repeats-13 (ADAMTS-13), and neurofilament (NF) in the brain tissue in small ruminants infected with Border Disease Virus (BDV) and to identify any correlation between hypomyelinogenesis and BD neuropathology. Results of the study revealed that the levels of ADAMTS-13 (*p*<0.05), nNOS (*p*<0.05), and NF (*p*<0.05) were remarkably higher in BDV-infected brain tissue than in the uninfected control. It was suggested that L-arginine-NO synthase pathway is activated after infection by BDV and that the expression of NF and nNOS is associated with the severity of BD. A few studies have focused on ADAMTS-13 expression in the central nervous system, and its function continues to remain unclear. The most prominent finding from our study was that ADAMTS-13, which contain two CUB domains, has two CUB domains and its high expression levels are probably associated with the development of the central nervous system (CNS). The results also clearly indicate that the interaction of ADAMTS-13 and NO may play an important role in the regulation and protection of the CNS microenvironment in neurodegenerative diseases. In addition, NF expression might indicate the progress of the disease. To the best of the authors’knowledge, this is the first report on ADAMTS-13 expression in the CNS of BDV-infected small ruminants.

## Introduction

Border Disease (BD), caused by *Pestivirus*, is characterised macroscopically by congenital anomalies in the central nervous system (CNS), such as hydranencephaly, cerebellar hypoplasia, kyphosis, scoliosis, brachygnathism, and arthrogryposis of the skeletal system [1–3]. In addition to these anomalies, hypoplasia have also been observed in the testis and the thymus of animals with this disease [4–6].

ADAMTS-13 (A Disintegrin And Metalloprotease with Thrombospondin type I repeats-13) cleaves ultra-large von Willebrand factor multimers into multimers with lower activity to reduce potential thrombotic activity and prevent microvascular thrombosis [7], [8]. ADAMTS-13 is synthesised mainly in the hepatic stellate cells of the liver [9], but it is also expressed in vascular endothelial cells [10] and a number of parenchymatous organs including the brain [11], [12].

Its CUB (Complement protein subcomponents C1r / C1s, urchin embryonic growth factor and bone morphogenetic protein 1) domains are characterised by animmunoglobulin-like structure [13], [14]. It is known that, with their ligand-binding potential, these domains contribute to protein-protein and protein-carbohydrate interactions, and in this way, these domains play a fundamental role in developmental processes such as embryogenesis and organogenesis [13–16]. A number of proteins that contain CUB domains also play a role in this process [13], but the ADAMTS-13 CUB domains are unique and are not found in any other ADAMTS or ADAM proteases [17–19]. However, the role of ADAMTS-13 in the CNS, and the systemic roles of its CUB domains have not been fully understood.

As well as its neuroprotective effects, the free radical nitric oxide (NO) may also have neuropathological effects on the CNS, depending on the intensity of its expression [20]. NO expressed at pathological levels has been shown to cause neuronal degeneration as a result of its neurotoxic effects [21–23]. Only one study has shown the contribution of endothelial nitric oxide synthase (eNOS) and inducible nitric oxide synthase (iNOS) to neuronal degeneration and endogenous apoptosis in the CNS of small ruminants infected with Border Disease Virus (BDV) [23]. However, there is no report indicating that pathological levels of NO are neuronal in origin in BDV infected animals.

The present study investigated the connection between ADAMTS-13 and foetal development and hypomyelinogenesis in the CNS, which is primarily affected in this disease, and in other organs that show developmental anomalies. We also examined whether the severity of the degeneration in the CNS occurring in the disease has any connection with neuronal nitric oxide synthase (nNOS). The severity of the degeneration was also assessed in terms of its neurofilament (NF) expressions in infected animals and healthy control groups.

## Materials and Methods

### Ethics Statement

Approval of Committee on the Ethics of Animal Experiments of the Kirikkale University was not required because the experiment did not involve any invasive procedures for animal experiment. The study specimens (5 kids and 10 lambs) were brought to Kırıkkale University Veterinary Faculty Department of Pathology for routine necropsy of died sheep, goats, and their aborted fetuses. All the examined animals had been submitted to the necropsy by the owners’ for routine diagnostic procedure. Thus, none of the animals was not sacrificed. The healthy control group animals (6 kids and 6 lambs), that had been slaughtered for human consumption, were collected from the Kirikkale Slaughterhouse. Only the heads of sacrifice of the animals were purchased.

### Pathologic Examination

The brain samples of the kids and lambs that are included in the study, confirmed as Border Disease Virus (BDV) positive both in reverse transcriptase polymerase chain reaction (RT–PCR) and immunoperoxidase tests. Healthy control group was constituted of the brain samples of slaughtered healthy lambs (n = 6) and kids (n = 6). The brains were removed and fixed in 4% paraformaldehyde in phosphate-buffered saline (PBS) at pH 7.4 for 48h and then were thoroughly rinsed overnight, under tap water. After performing the routine tissue preparation procedures of dehydration using graded alcohol and xylene, the tissue samples were embedded in paraffin blocks; 4–5 μm-thick paraffin sections were then cut and mounted on glass slides. Hematoyxlin-Eosin (H&E) and immunohistochemicaltests were performed, andthey were analyzed using a trinocular light microscope (Olympus BX51 and DP25 digital camera). The severity of BDV infection in each animal was classified according to neuropathological changes as follows; gliosis, hypomyelinization, viral antigen immunopositivity in the neurons, endothelial and glial cells.

### Immunoperoxidase Examinations

Immunohistochemistry was performed to observe ADAMTS-13, nNOS, and NF in the 4–5 μm-thick paraffin sections of the tissues by using an indirect streptavidin/biotin immunoperoxidase kit (HRP, ThermoScientific, USA), as per the manufacturer’s instructions. Briefly, the sections were placed onto adhesive slides, deparaffinized for 5min. Each in the 3-step xylene series, and rehydrated using a series of graded alcohol and distilled water. The antigens were retrieved by boiling the tissue sections on glass slides in citrate buffer (pH 6.0) (Thermo Scientific, USA) for 20 min. Endogenous peroxidase activity was quenched using 3% hydrogen peroxide in absolute methanol for 7 min at room temperature (RT). The tissue sections were rinsed thrice with PBS (pH 7.4) for 5min, between each consecutive step. The sections were then incubated in a blocking serum for 5min to prevent non-specific antibody binding. Thereafter, the sections were incubated with a primary antibody (ADAMTS-13, nNOS, and NF) for 60 min in a humidity chamber at the RT. After treating the sections with biotin-labeled secondary antibody for 15min and streptavidin-peroxidase enzyme for 15 min at RT, the color reaction was performed using aminoethylcarbasole (AEC) chromogen (Thermo Scientific, USA) for 5–10 min. Sections were counterstained with Mayer’s hematoxylin for 1–2min and suspended in water-based mounting medium (Thermo Scientific, USA). For each immunoperoxidase test, two negative and positive control tissue sections were allowed as follows; as a positive control: mouse forebrain and spinal cord for nNOS, mouse liver tissue for ADAMTS-13 and brain section for NF. As negative control, the one of the serial parafin sections incubated with normal mouse serum (isotype serum control) instead of primary antibody. Additionally, to control non-specific endogenous peroxidase and biotin activities in each test, the primary antibody step was omitted.

### Luxol Fast Blue Staining

For myelin evaluation, Luxol Fast Blue (LFB, a demyelination marker) staining of the 4–5μm thick cross-sections was performed. The paraffin sections were deparaffinized, rehydrated, and incubated with 1% LFB stain overnight at 57°C in tightly sealed staining jars. These sections were then hydrated with 95% and 75% alcohol, differentiated by several changes of 70% ethanol and 0.05% lithium carbonate (LiCO3) solution for few seconds. Thereafter, the sections were washed thoroughly with distilled water, counterstained at 60°C in pre-heated 1% cresyl violet acetate, containing 0.1% acetic acid (pH 3.7) cresyl-violet. The volume of the demyelinated region was calculated using the automated histomorphometry analysis software (Leica, Germany). The brain tissue cross-sections were identified based on the following characteristics: areas of normal myelin appeared dark bright blue and the demyelinated tissue appeared blanched.

### Antibodies

Commercial, mouse antibodies against anti-bovine viral diarrhea viruses 1 and 2 (VMRD, Inc. cat. 157) diluted to 1:250, ADAMTS-13 (Abcam, Cambridge, UK) diluted to 1:100, nNOS (Santa Cruz Biotechnology, USA) diluted to 1:100, and undiluted NF (Thermo Scientific, USA), were used. This commercially available (Catalog no: 157) primary antibody has given cross reactivity with BDV.

### Histomorphometric Analysis and Statistics

For statistical evaluation of the intracellular and interstitial immunostaining, Leica QWin Plus image processing and automated histomorphometry analysis software (Leica, Germany) was used. Briefly, at least 5 randomly selected and consecutive 20x objective microscopic fields were photographed (Lecia DM4000 B). After calculating the proportion (% pixels) of stained area to the whole field, the mean (in % pixels) staining area for each slide was determined. For evaluating the non-parametric data, Mann-Whitney U-test was performed to compare ADAMTS-13, nNOS, and NF immunoreactive cells and immunopositively stained areas in the animals infected with BDV versus the healthy controls. A *p* value of <0.05 and <0.005 was considered significant. For the statistical analyses, MS-Excel 2003 and SPSS for Win. Ver. 15.0 (SPSS Inc., Chicago, IL, USA) programs were used.

## Results

### Gross Lesions and Histopathologic Findings

Necropsy of fetal and neonatal brain samples helped detect cerebellar hypoplasia, porencephaly, and subcortical cavitations in the cerebral cortex, in addition to hydrocephalus. Additionally, arthrogryposis, scoliosis, brachygnathia inferior, and gingival hypertrophy were also observed.

**Fig 1:**
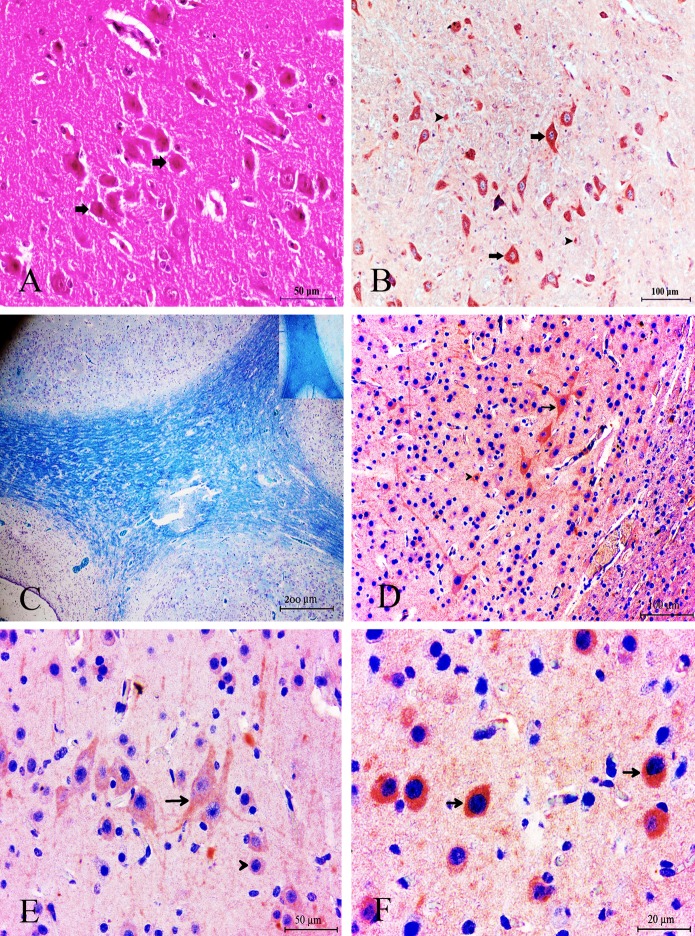
Histological section of brain tissue was that widespread observing shrinkage, degenerations and necrosis in the neurons (arrows). H&E, Bar = 50 μm (A) Immunohistochemical detection of intense BDV antigen. Note the positive immunolabelling (red pigment) in degenerative/necrotic neuron cytoplasm (arrows) and glial cells (arrowhead). ABC technique (anti-BVD), Mayer's hematoxylin counterstain, Bar, 100 μm (B) Severe hypomyelination, the myelin site was not painted at all (arrow). Positive control (small photo in the corner). LFB, Bar = 200 μm (C) Strong expression of ADAMTS-13 in glial (arrowhead) and neuronal cells (arrow). ABC technique (anti-ADAMTS-13), Mayer's hematoxylin counterstain, Bar, 100 μm (D) Strong expression of ADAMTS-13 in glial (arrowhead) and neuronal cells (arrow). ABC technique (anti-ADAMTS-13), Mayer's hematoxylin counterstain, Bar, 50 μm (E) Strong expression of ADAMTS-13 in neuronal cells (arrow). ABC technique (anti-ADAMTS-13), Mayer's hematoxylin counterstain, Bar, 20 μm (F).

Microscopic lesions were observed in the brains of all the animals. The most conspicuous findings were those of hypomyelinogenesis in all parts of the brain along with nonsuppurative lymphohistiocytic meningoencephalomyelitis characterized by severe gliosis and perivascular mononuclear infiltration. The cerebral cortices showed thinning and were composed of glial cells, necrotic neurons, and vessels (Fig. 1A). The normal laminar arrangement of neurons and glial cells was not observed. Central chromatolysis and neuronal necrosis were also observed in the brain.

### Immunoperoxidase Findings

We analyzed the protein expression levels of ADAMTS-13, nNOS, and NF in the brain tissues from BDV-infected and healthy control animals. Immunohistochemical analysis showed significant up-regulation of ADAMTS-13, nNOS, and NF expression in the BDV-infected animals unlike that in case of the healthy control animals (Table A and B in S1 File). Statistical analysis of the data on ADAMTS-13, nNOS, and NF expressions in the brain, measured by immunostaining in all the groups, are listed in Table C in S1 File.

### Distribution of Viral Antigens in the Brain Sections

Neurons of both the cerebellum and cerebrum showed strong staining for diffused BDV antigen (Fig. 1B). In addition, intense staining was observed in the vasculature of the meninges overlying the cerebrum and cerebellum in all the animals.

### LFB Findings

BDV-infected animals showed significantly blanched areas of demyelination, while high intensity staining was evident in the healthy-control group (Fig. 1C). LFB staining revealed significant demyelination in the white matter of the cerebrum and cerebellum in all the BDV-infected animals.

### ADAMTS-13 and Neuronal Nitric Oxide Synthase Expression

We measured the protein expression of ADAMTS-13 and nNOS in all parts of the brain. ADAMTS-13 and nNOS expression increased significantly within and in the periphery of the lesion, which was also significantly higher than the levels in the control group (*p*<0.05). Fairly weak nNOS expression was observed in neurons, endothelial and glial cells in healthy animals (healthy control group) (Table A and Table B in S1 File). However, the intensity of nNOS expression in the brain tissue of BDV-infected animals was detected to be more severe than healthy control group. This assessment has also been interpreted quantitatively and difference was statistically significant (p<0.05).

**Fig 2:**
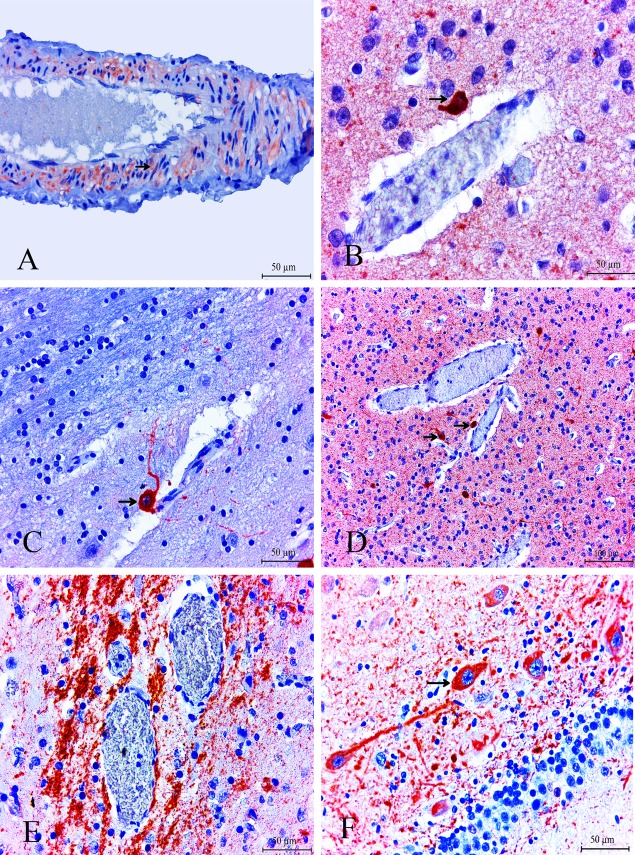
nNOS expression was markedly increased in the endothelial cells (arrow). ABC technique (anti-nNOS), Mayer's hematoxylin counterstain, Bar, 50 μm (A) Strong expression of nNOS in neuronal cells was noted in areas surrounding the cerebral blood vessels ABC technique (anti-nNOS), Mayer's hematoxylin counterstain, Bar, 50 μm (B) Strong expression of nNOS in neuronal cells was noted in areas surrounding the cerebral blood vessels ABC technique (anti-nNOS), Mayer's hematoxylin counterstain, Bar, 50 μm (C) Strong expression of nNOS in neuronal cells was noted in areas surrounding the cerebral blood vessels ABC technique (anti-nNOS), Mayer's hematoxylin counterstain, Bar, 100 μm (D) Massive, regional accumulation of NF was noted in areas surrounding the cerebral blood vessels. ABC technique (anti-NF), Mayer's hematoxylin counterstain, Bar, 50 μm (E) Abnormal massive accumulation of neuronal NF was detected in the BDV-infected animal brains ABC technique (anti-NF), Mayer's hematoxylin counterstain, Bar, 50 μm (F).

ADAMTS-13 was expressed by some glial (Fig. 1D) and endothelial cells in the cerebral cortex but was predominantly expressed by the neurons (Fig. 1D,E,F). The most conspicuous finding of the present study was that nNOS expression was markedly increased in the endothelial cells (Fig. 2A) and in areas surrounding the cerebral blood vessels (Fig. 2B,C,D).

BDV-infected animal brains showed enhanced levels of nNOS and ADAMTS-13, and prolonged release of NO, which may contribute to neurotoxicity and parenchyma degeneration. This may be responsible for the severity of the disease and the permeability of the blood-brain barrier.

### Neurofilament (NF) Expression

In this study, neurofilament accumulation and axonal degeneration were observed in brain tissue of animals infected with BDV. Massive, regional accumulation of NF was noted in areas surrounding the cerebral blood vessels (Fig. 2E). Abnormal massive accumulation of neuronal NF was also detected in the BDV-infected animal brains (Fig. 2F). Moreover, sites of enhanced NF immunoreactivity were colocalized inside the lesion (demyelinated and focal gliosis area), and this phenomenon was significantly more pronounced in BDV-infected animals than in the healthy-control group (*p*< 0.05).

## Discussion

A few studies have investigated the expression of ADAMTS-13 in the central nervous system [12], [24], [25]. A study carried out by Tauchi et al. in 2012 showed that ADAMTS-13 was expressed in the glial cells, but not in the neurons. In the present study, in contrast, it was observed that ADAMTS-13 was particularly expressed in the neurons of sheep and goats infected with BDV. Moreover, this is the first study to show the expression of ADAMTS-13 in the brain tissues of small ruminants infected with BDV. It was also stated earlier that high NO expression caused degeneration of the brain tissues of animals infected with BDV [23]. In the present study, nNOS was also shown to be a source of NO, and the severity of the associated degeneration in the CNS was shown in terms of expression of NF.

Nitric oxide has a protective effect on neurons at normal physiological levels, but it exhibits neurotoxic effects at high concentrations in association with other free radicals [20]. Thus, the necrosis of neurons may be caused by high NO expression [21], [22]. In animals infected with BDV, the neurotoxic effects of NO expressed at high levels may be seen in macrophages, which have infiltrated the meninges, neurons, and glial and endothelial cells and by CNS degeneration [23]. In the same study, it was also shown that iNOS was not exclusively responsible for this effect, buteNOS may also be involved [23]. In the present study, it was shown that the neuronal expression of NO (as well as of iNOS and eNOS) may also be responsible for the degeneration and necrosis in the CNS. This means that BDV infection results in high NO expressionin the CNS. It is concluded that due to its neurotoxic effects when expressed at pathological levels, NO causes degeneration and neuronal necrosis.

Nitric oxide increases fluid shear stress, and eNOS makes the greatest contribution to this high shear stress [26], [27] arising in the narrow vessels triggers vWF-mediated platelet adhesion and aggregation after vessel injuries [28], [29]. After tissue damage has occurred, ADAMTS-13 cleaves von Willebrand factor (vWF) in microcirculation regions and during thrombus formation [30–32]. eNOS expression was observed in the glial cells, neurons, and especially in the endothelial cells of aborted sheep and goat foetuses infected with BDV, whereas iNOS expression was observed in gliotic foci, macrophages and especially in the glial cells surrounding the blood vessels [23]. In the present study, in addition to these findings, it was observed that in animals infected with BDV, there was a significantly high nNOS expression in neurons and endothelial cells. It was also observed that these neurons were mainly in areas close to blood vessels. Additionally, the fact that ADAMTS-13 and NO were expressed at levels that were statistically higher in BDV infected animals than in control groups indicates that the fluid shear stress was considerably elevated in these animals, especially in the aborted foetuses.

The blood-brain barrier (BBB) is a physical and metabolic barrier that regulates and protects the microenvironment in the CNS and separates the CNS from the peripheral circulation [33]. The permeability of the BBB is increased in BD. This facilitates the movement of activated leukocytes and the virus into the brain parenchyma and the perivascular gap. This process plays a critical role in promoting rapid infection of the CNS cells and in triggering and aggravating inflammation. It has been observed that the permeability of the BBB was significantly increased in mice in which vWF, which is closely associated with ADAMTS-13, was inhibited [34]. Impairment of tight junctions, which play a role in the transmembrane protein interactions between the BBB and the endothelial cells, also increases the permeability of the BBB in diseases characterised by myelin disorders such as multiple sclerosis and idiopathic inflammatory demyelination [33]. On the basis of this information, it was concluded that ADAMTS-13 is closely related to the formation of myelin and that it may cause changes in BBB permeability by cleaving not only the vWF, but also other transmembrane proteins. It is concluded from the present study that in infections in animals which have completed organ development (infections in the late stages of pregnancy), ADAMTS-13 was strongly expressed in order to suppress the negative effects of the virus and that it contributed to the reduction in the permeability of the BBB. It is therefore anticipated that knowledge of the effects of ADAMTS-13 on the permeability of the BBB and the role of such effects in demyelination will help understand the pathogenesis of BD and the control of the disease.

Accumulation of NF at pathological levels is an important sign in the diagnosis of acute parenchymal destruction observed in neurodegenerative and cerebral diseases, because NF accumulation is seen in damaged neurons [35–37]. Thus, by looking at the quantity of NF in the cerebrospinal fluid, an opinion can be reached about the severity of the degeneration [35]. High GFAP [glial fibrillary acidic protein] expression in animals infected with BDV are also indicative of CNS degeneration [23]. In the present study, significantly high NF expression in the neurons, axons, and parenchymal tissue of animals infected with BDV was observed. It is thus seen that there was parenchymal degeneration associated with gliosis and neuronal necrosis. On the basis of the findings of this study, it may be suggested that the diagnosis in live animals infected with BDV may be further strengthened by examining the level of NF in the cerebrospinal fluid in these animals, which varies in intensity at different stages of pregnancy. We also believe that this may give information about the degree of neuronal degeneration. It is thought that this clinical approach may also be a model for diagnosis and subsequent treatment of other CNS infections and degenerative illnesses.

Thrombospondin type 1 repeats (TSRs) are found in extracellular proteinsand play a functional role in intercellular and cell-matrix interaction and signalling. Thrombospondin 1, properdin, F-spondin, ADAMTS-13 and ADAMTSL1 are proteins that contain TSRs. The common property of these proteinsis that they have important functions in embryonic development, tissue formation, angiogenesis, neurogenesis, and complement activation [13], [38]. In view of the macroscopic symptoms, which are characteristic of *Pestivirus* infections, such as hydranencephaly, cerebellar hypoplasia, as well as hypomyelinogenesis, it is considered that ADAMTS-13, which contains a CUB domain and more than seven TSRs, may play a role in the development of the foetus.

In conclusion, it is believed that ADAMTS-13 plays a significant role in the development of the cerebrum and cerebellum during the embryonic stage. In addition, it was seen that not only the NO synthesized by eNOS and iNOS, but also the NO synthesized by nNOS, which is strongly expressed in neurons and endothelial cells, plays a role in neuronal degeneration, gliosis, and necrosis in BD. It is thought that in CNS degeneration in animals infected with BDV, NO accumulation may be a result of the pathological process rather than an etiological factor. Subsequent to these findings, it is believed that NF may be important in identifying the severity and the disease progression during follow-up, and NF levels may also be used clinically. It is also concluded that the knowledge of the connections between BD and the biosynthesis, expression, and inhibition of ADAMTS-13 will be of significance in identifying the pathogenesis of the disease and in the development of therapeutic protocols.

## Supporting Information

S1 File Table A. Calculating the proportion (% pixels) of ADAMTS-13, nNOS and NF stained area to the whole field activitys in lambs. *Table B*. Calculating the proportion (% pixels) of ADAMTS-13, nNOS and NF stained area to the whole field activitys in kids. *Table C*. Immunoperoxidase test results and statistical data.
(PDF)
