# Supplementary material for: Increased Expressions of ADAMTS-13, Neuronal Nitric Oxide Synthase, and Neurofilament Correlate with Severity of Neuropathology in Border Disease Virus-Infected Small Ruminants
Source: PLoS One. 2015 Mar 23;10(3):e0120005. doi: 10.1371/journal.pone.0120005 (PMC4370801; doi:10.1371/journal.pone.0120005)
Supplement: S1 File — Calculating the proportion (% pixels) of ADAMTS-13, nNOS and NF stained area to the whole field activitys in lambs. Table B. Calculating the proportion (% pixels) of ADAMTS-13, nNOS and NF stained area to the whole field activitys in kids. Table C. Immunoperoxidase test results and statistical data. (PDF) [file pone.0120005.s001.pdf]

Table A. Calculating the proportion (% pixels) of ADAMTS-13, nNOS and NF stained area to the whole field activitys in lambs

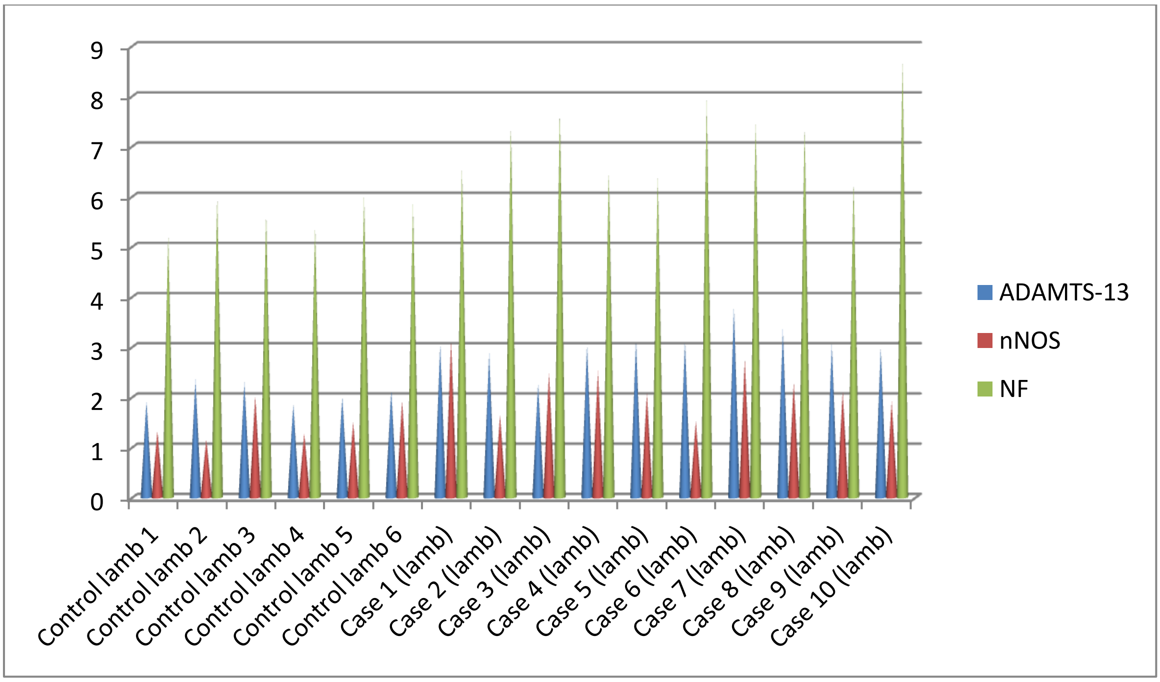

Table B. Calculating the proportion (% pixels) of ADAMTS-13, nNOS and NF stained area to the whole field activitys in kids

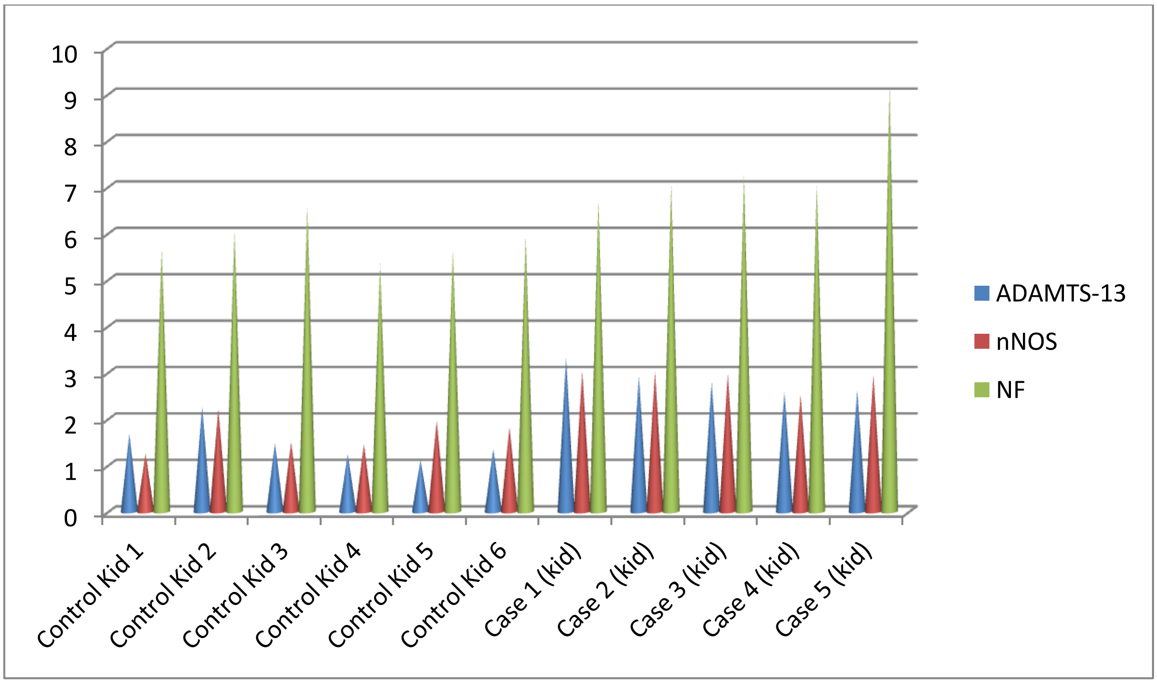

Table C. Immunoperoxidase test results and statistical data

ANIMALS	N	ADAMTS-13		*p*	nNOS		*p*	NF		*p*
		Mean	Sd		Mean	Sd		Mean	Sd	
Control lambs	6	2.186	0.237	0.028	1.501	0.458	0.043	5.614	0.388	0.011
BDV positive lambs	10	3.060	0.380		2.244	0.499		7.245	0.791	
Control kids	6	1.799	0.408	0.025	1.655	0.501	0.025	6.103	0.453	0.025
BDV positive kid	5	2.844	0.296		2.901	0.216		7.455	0.999	
Control small ruminants	12	1.993	0.366	0.001	1.578	0.438	0.004	5.858	0.463	0.001
BDV positive small ruminants	15	2.988	0.359		2.463	0.526		7.315	0.836	
